# Elevated plasma matrix metalloproteinases associate with *Mycobacterium tuberculosis* blood stream infection and mortality in HIV-associated tuberculosis

**DOI:** 10.1093/infdis/jiae296

**Published:** 2025-02-04

**Authors:** NF Walker, C Schutz, A Ward, D Barr, C Opondo, M Shey, PT Elkington, KA Wilkinson, RJ Wilkinson, G Meintjes

**Affiliations:** 1 Department of Clinical Sciences, Liverpool School of Tropical Medicine, Liverpool, L3 5QA, United Kingdom; 2 Centre for Infectious Diseases Research in Africa, Institute of Infectious Disease and Molecular Medicine, University of Cape Town, Observatory 7925, South Africa; 3 TB Centre and Department of Clinical Research, London School of Hygiene and Tropical Medicine, WC1E 7HT, United Kingdom; 4 Department of Medicine, University of Cape Town, Observatory 7925, South Africa; 5 Wellcome Liverpool Glasgow Centre for Global Health Research, University of Liverpool, Liverpool, L69 3BX, United Kingdom; 6 Department of Infectious Diseases, Queen Elizabeth University Hospital, Glasgow, G51 4TF; 7 Department of Medical Statistics, London School of Hygiene and Tropical Medicine, WC1E 7HT, United Kingdom; 8 NIHR Biomedical Research Centre, School of Clinical and Experimental Sciences, Faculty of Medicine, University of Southampton, Southampton, SO16 6YD, United Kingdom; 9 The Francis Crick Institute, London, NW1 1AT, United Kingdom; 10 Department of Infectious Diseases, Imperial College London, W12 0NN, United Kingdom

**Keywords:** Tuberculosis, HIV, mortality, matrix metalloproteinase

## Abstract

Mortality from HIV-associated tuberculosis (HIV-TB) is high, particularly among hospitalised patients. In 433 people living with HIV hospitalised with symptoms of TB, we investigated plasma matrix metalloproteinases (MMP) and matrix-derived biomarkers in relation to TB diagnosis, mortality and *Mycobacterium tuberculosis* (*Mtb*) blood stream infection (BSI). Compared to other diagnoses, MMP-8 was elevated in confirmed TB and in *Mtb*-BSI, positively correlating with extracellular matrix breakdown products. Baseline MMP-3, -7, -8, -10 and PIIINP associated with *Mtb*-BSI and 12-week mortality. These findings implicate MMP dysregulation in pathophysiology of advanced HIV-TB and support MMP inhibition as a host-directed therapeutic strategy for HIV-TB.

## Background

Tuberculosis (TB) is a leading infectious cause of death worldwide, resulting in an estimated 1.3 million deaths annually (1). The End TB Strategy has highlighted prevention of TB deaths as a major target and aims to reduce TB deaths by 95% between 2015 and 2035 (2). In people living with HIV, TB is the leading cause of death (3). To meet End TB targets, identification of and interventions for those at highest risk of poor outcomes are needed, particularly for use in low-resource settings where the burden of TB falls most heavily. Further understanding of the causes of mortality and pathophysiology of TB disease is required to prevent TB deaths in people living with HIV.

We have previously reported that plasma matrix metalloproteinase (MMP)-1, MMP-8 (neutrophil collagenase) and procollagen N-terminal propeptide (PIIINP), a matrix degradation product released during collagen turnover, are elevated in patients with active TB compared to patients without TB, highlighting collagen turnover as a feature of TB disease, in both HIV positive and negative cohorts (4, 5). Plasma PIIINP was significantly higher in HIV positive patients with newly diagnosed active TB compared to HIV negative patients, positively correlated with HIV viral load and was elevated during TB immune reconstitution inflammatory syndrome (IRIS) (4).

Here, we evaluated plasma MMP, PIIINP and extracellular matrix components, collagen type IV alpha 1 chain (Col4α1) and hyaluronic acid (HA), in a cohort of patients hospitalised with advanced HIV and TB symptoms, thereby focusing on the population most requiring interventions to reduce mortality. We aimed to evaluate potential as diagnostic biomarkers and hypothesised that disseminated *Mycobacterium tuberculosis* (*Mtb*) in HIV-TB may drive systemic MMP upregulation and consequently tissue damage. We report a novel association of elevated plasma MMP with *Mtb*-blood stream infection (BSI) and mortality in advanced HIV-TB, providing pathophysiological insights.

## Methods

The study was approved by the University of Cape Town Human Research Ethics Committee (HREC ref 057/2013) and London School of Hygiene and Tropical Medicine Research Ethics Committee (ref 11710). Full methods have been reported elsewhere (6). Eligible patients were adults with HIV infection and a CD4 count ≤ 350 cells/μl, admitted to Khayelitsha Hospital, Cape Town, with a probable new diagnosis of TB. Exclusion criteria were pregnancy, TB treatment within one month prior to admission or more than 3 doses of TB treatment prior to enrolment, or unknown HIV status (declined testing). Participants provided written informed consent. Eligible patients with decreased capacity to consent were enrolled and followed up daily to obtain consent according to the approved protocol. Participants were investigated by TB culture (sputum, blood), Xpert (sputum, urine) and urine lipoarabinomannan (LAM, Alere Determine TB LAM assay) prior to initiation of TB treatment. All results were made available to the clinical team and participants remained in routine clinical care. Vital status was determined at 12 weeks.

Participants were classified retrospectively as microbiologically **confirmed TB** if *Mtb* was identified in clinical samples, **clinical TB** if TB was likely and treatment for TB was given following WHO guidelines but no microbiological confirmation was obtained, **no TB**, if TB was excluded on clinical and microbiological grounds, or **LAM TB** if criteria for no TB was met but urine LAM was positive >=2 by two independent readers (6).

Inclusion in this analysis required the availability of EDTA plasma samples at enrolment. MMP -1, -3, -7, -8, -9 and -10 were quantified by Luminex array (Bio-Rad Bio-Plex 200 system; assay R&D Systems, United Kingdom). PIIINP, Col4α1 and HA were quantified by enzyme-linked immunosorbent assays (Cloud Clone Corp, China), as per manufacturers’ instructions. HA and Col4α1 measurement were limited to a subset of 73 randomly selected participants. Statistical analysis was performed in Prism 9 and R Studio (2023.03.1). Comparisons of analytes by TB diagnosis was between no TB and either confirmed TB or clinical TB and a Bonferroni correction was performed as two groups were compared for one analyte. Statistical significance was inferred by a p value <0.05. Comparisons between two groups were by Mann-Whitney U test unless otherwise stated.

The relationship between MMP or PIIINP concentrations and *Mtb*-BSI or mortality was depicted using a Loess non-parametric smoother and assessed using a mixed effects model, including a random effect on intercept for plate, to account for any technical variation between plates as analytes were measured across multiple plates. Hierarchical clustering analysis was performed by Ward’s method based on Euclidean distance, to assess the association between analytes, including neutrophil count, neutrophil percentage and procalcitonin, but excluding Col4α1 and HA, as they had only been measured in a subset of patients on scaled data (mean subtracted and divided by standard deviation), excluding extreme outliers (observations with a value greater than four median absolute deviations from the variable median applied after transformation). Pearson correlation to assess the association between analytes was performed on log-transformed values, excluding extreme outliers as above.

## Results

### Participant demographics, diagnoses and outcomes

Plasma samples were available for 437 participants (Supplementary Table S1). TB diagnosis was confirmed in 313 (71.6%) and clinical TB in 48 (11.0%). Only 4 participants met the criteria for LAM TB, so these were excluded from subsequent analyses. In no TB (n=72, 16.5%), community acquired pneumonia (n=34, 47.2%) was the most frequent diagnosis, followed by other blood pathogen (n=8, 11.1%), *Pneumocystis jirovecii* pneumonia (n=6, 8.33%) and cryptococcal disease (n=6, 8.33%). The median CD4 count was 56 cells/mm^3^ (IQR 18.0-112) in confirmed TB, 93 (IQR 53.8-182) in clinical TB and 89.5 (IQR 29.0-224) in no TB.

Vital status at 12 weeks was known for 427/433 (98.6%) participants. Death occurred in 82/433 (18.9%) participants at a median of 16.0 (IQR 2.75-44.3) days. Mortality at 12 weeks in confirmed TB was 19.8% (62/313). *Mtb*-BSI was present in 133/313 (42.5%) participants with confirmed TB and was associated with increased mortality: 37/133 (27.8%) participants with *Mtb*-BSI died compared to 23/173 (13.3%) without (p = 0.002 by Fisher’s Exact Test). Participant characteristics are reported by vital status and *Mtb*-BSI in Supplementary Table S2. More *Mtb*-BSI occurred in men than women, but there was no difference in mortality. Older age was associated with mortality, but not *Mtb*-BSI. Being ART naïve or having defaulted was associated with increased *Mtb*-BSI, compared to being ART treated. *Mtb*-BSI and mortality were associated with lower CD4 counts.

### Plasma MMP-8 is elevated in patients hospitalised with HIV-associated TB

**Figure 1:**
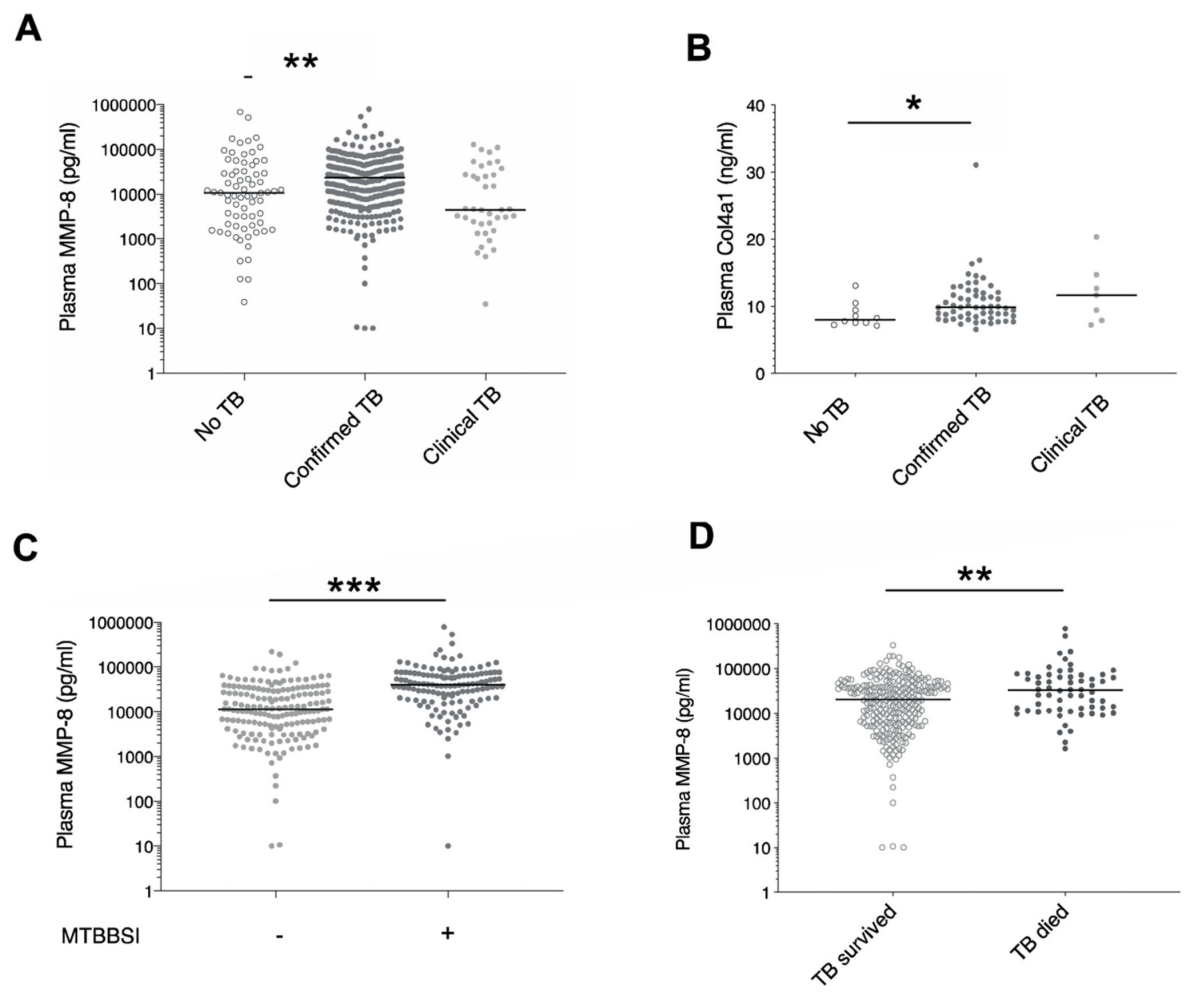
Elevated matrix metalloproteinase-8 in HIV-TB, *Mtb*-blood stream infection and TB mortality Plasma matrix metalloproteinase (MMP)-8 (A) and Col4α1 (B) were elevated in hospitalised participants with HIV infection and microbiologically-confirmed TB, compared to hospitalised HIV positive participants with symptoms due to other diagnoses (no TB). Differences between confirmed TB and TB diagnosed clinically were not statistically significant. Plasma MMP-8 was increased in participants with confirmed TB and *Mycobacterium tuberculosis* blood stream infection (MTBBSI) compared to those without (C) and in those who had died compared to those who had survived at 12 weeks (D). Col4α1 was measured in a subset of 73 participants. *p<0.05, **p<0.01, ***p<0.001.

We first examined plasma MMP and extracellular matrix breakdown product concentrations in no TB in comparison to confirmed TB or clinical TB. Plasma MMP-8 was significantly elevated in confirmed TB compared to no TB (median 23712 pg/ml, IQR 7688-47571 vs median 10602, IQR 2019-32205; p=0.003; Figure 1A and Supplementary Table S3), as was plasma Col4α1 (Figure 1B). Plasma MMP-3 and -10 were lower in participants with confirmed TB compared to no TB and clinical TB, whilst MMP-1, -7 and -9, PIIINP and HA did not differ between groups. These findings were similar in male and female participants (Supplementary Figure S1). MMP-8 did not differ significantly by sex (Supplementary Table S4).

### Plasma MMP-8 is associated with Mtb blood stream infection and mortality in HIV-associated TB

MMP are primarily transcriptionally regulated however, MMP-8 may be stored in neutrophil granules. Exploring the hypothesis that disseminated *Mtb* drives MMP-8 upregulation and release from neutrophils and that this associates with poor outcomes in HIV-TB, we next examined MMP-8 concentration in the presence or absence of *Mtb*-BSI and by vital status at 12 weeks. We found that in confirmed TB, those with *Mtb*-BSI had elevated plasma MMP-8 (Figure 1C) compared to those without (median 40003 pg/ml, IQR 20006-70583 vs median 11451, IQR 4697-30789; p<0.001). Plasma MMP-8 was elevated in participants with confirmed TB who died compared to those who survived (median 32811 pg/ml IQR 12060-66934 vs median 20201, IQR 6050-40561, p=0.002, Figure 1D). Col4α1 and HA concentrations did not differ by vital status (data not shown), although Col4α1 was elevated in confirmed TB with *Mtb-*BSI compared to without (median 11.2 ng/ml, IQR 7.76-9.92 vs median 8.75 IQR 9.76-13.5, p<0.001). Plasma MMP-8 positively correlated with Col4α1 (Pearson r = 0.535, p<0.001) and PIIINP (Pearson r = 0.404, p<0.001).

### Multiple MMP and PIIINP associate with HIV-TB severity

**Figure 2:**
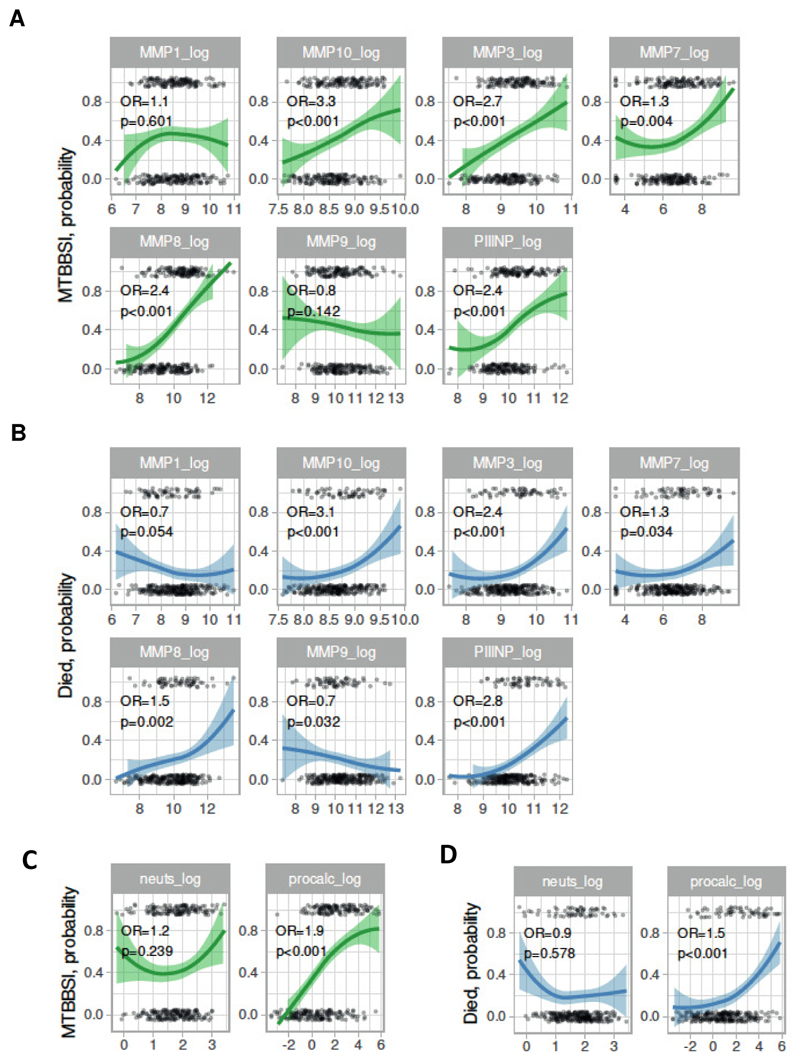
Plasma matrix metalloproteinase (MMP) and procollagen III N-terminal propeptide (PIIINP) associate with *Mycobacterium tuberculosis* blood stream infection and mortality in HIV-associated TB. Probability (dependent variable, y axis) of *Mycobacterium tuberculosis* blood stream infection (MTBBSI, A/C) and mortality (B/D) by analyte log concentration (predictor variable, x-axis). Loess fit to data (coloured lines with shaded 95% confidence intervals) demonstrates the functional relationship, with measured concentrations shown as a jittered scatterplot at 0 (survived/no MTBBSI) or 1 (died/MTBBSI) on the y axis. Odds ratios (OR) and 95% confidence interval (CI) are from logistic regression models, with adjustment for random effects by plate. In A and B, log MMP or PIIINP concentrations are in pg/ml. In C/D, log neutrophil count is x10^9^/L and log procalcitonin concentration in μg/L.

We proceeded to examine the association of other plasma MMP and PIIINP with *Mtb*-BSI and mortality in confirmed TB. We found a positive association between plasma MMP-3, -7, -8, -10 and PIIINP for *Mtb*-BSI and mortality (Figure 2A-B). We performed hierarchical clustering analysis including neutrophil count, neutrophil percentage, and procalcitonin as biomarkers of acute inflammation, MMP concentrations and PIIINP (Supplementary Figure S2). MMP-8 most closely clustered with PIIINP and procalcitonin. Procalcitonin, but not neutrophil count, positively associated with *Mtb*-BSI and mortality (Figure 2C-D).

## Discussion

Patients with advanced HIV-1 hospitalised with symptoms of TB are at high risk of mortality. We found that baseline plasma MMP-8 and Col4α1 were increased in hospitalised patients with HIV infection who were confirmed to have TB compared to those who eventually received an alternative diagnosis. Contrary to our previous finding in outpatients, in this hospitalised cohort, plasma PIIINP was not elevated in patients with TB compared to symptomatic patients with alternative diagnoses.

Elevated plasma MMP-8, together with MMP-3, -7, -10 and PIIINP associated with *Mtb*-BSI and mortality at 12 weeks, suggesting that MMP upregulation and matrix turnover are features of TB disease severity. This is in keeping with our recently reported finding that elevated plasma MMP-8 concentrations at the end of TB treatment are associated with persistent *Mtb* culture positivity (7). MMP-8 was most closely associated with procalcitonin (an acute phase reactant) and the matrix degradation product, PIIINP (released during Type III collagen turnover), suggesting that MMP-8 activity, collagen turnover and acute inflammation are closely related processes in HIV-TB (5, 8). HA, a polysaccharide component of the extracellular matrix which is released during extracellular matrix turnover has previously been found to predict AIDS events or death in HIV positive patients commencing antiretroviral therapy (9). Plasma HA was not associated with mortality in this study.

Whilst we have demonstrated an association between plasma MMP and mortality in HIV-TB, our study does not prove a causal relationship. Elevated MMP may be a function of *Mtb* bacterial load or *Mtb* dissemination, which is itself associated with mortality. *In vitro*, virulent *Mtb* is able to induce neutrophil-derived MMP-8 secretion, via an NF-kB-dependent mechanism, so it is possible that blood stream *Mtb* directly stimulates neutrophil MMP-8 release (10). However, elevated MMP-8 may also occur via upregulation of pro-inflammatory cellular networks and the finding that MMP-8 associated more closely with procalcitonin than neutrophil count or percentage consistent with networks upregulating MMP-8 (11). Increased MMP-8 in TB was positively associated with PIIINP, a matrix degradation product released during type III collagen turnover and Col4α1, a component of type IV collagen. MMP inhibition with doxycycline has been shown to be safe and effective for TB in HIV negative patients (12). Our results support the case for clinical trials of MMP inhibition with doxycycline as a host-directed therapy for HIV-associated TB.

Prior studies have examined MMP-8 concentrations in patients with TB, predominantly in those who are HIV negative (11). Our finding of elevated plasma MMP-8 in participants with confirmed TB compared to those symptomatic with other illnesses is consistent with previous reports in outpatients, although this is the first report in people who are HIV positive and hospitalised (4, 5, 13, 14). MMP-8 is emerging as a biomarker that may help identify patients with active TB, in some settings if used in combination with other screening tools. Here, we also report elevated plasma Col4α1 in HIV-TB and elevated Col4α1 was also associated with *Mtb*-BSI. Col4α1 is a subunit of Type IV collagen, a key component of basement membranes that has not previously been studied in human TB, to our knowledge.

A strength of this study is the combination of rigorous clinical and mycobacterial analyses that was employed to determine TB status. A limitation pertains to the use of a single blood culture to determine the presence or absence of *Mtb*-BSI. It is likely that sequential blood cultures would have a higher diagnostic accuracy for *Mtb*-BSI (15). We have not adjusted for known predictors of mortality in our analysis, as causal pathways are not well understood.

In summary, in hospitalised people living with HIV, with a clinical syndrome compatible with TB, MMP-8 was elevated in confirmed TB, likely due to *Mtb*-driven tissue damage, compared to other diagnoses. Furthermore, plasma MMP-3, -7, -8, -10 and PIIINP associated with *Mtb*-BSI and mortality at 12 weeks, implicating MMP dysregulation in HIV-TB morbidity. MMP inhibition is a potential therapeutic target for this patient group, who are at urgent need of improved therapeutic strategies.

## Supplementary Material

Supplementary Materials
