# Supplementary material for: Elevated plasma matrix metalloproteinases associate with Mycobacterium tuberculosis blood stream infection and mortality in HIV-associated tuberculosis
Source: J Infect Dis. Author manuscript; Available in PMC 2024 Nov 18. (PMC7616822; doi:10.1093/infdis/jiae296)
Supplement: Supplementary Materials [file EMS200021-supplement-Supplementary_Materials.docx]

**Elevated plasma matrix metalloproteinases associate with *Mycobacterium tuberculosis* blood stream infection**

**and mortality in HIV-associated tuberculosis**

Supplementary Tables and Figures

**Supplementary Table S1 Demographic and Clinical Features of Study Participants**

|  | **Non-TB** | **Confirmed TB** | **Clinical TB** | **LAM TB*** | **All participants** |
| --- | --- | --- | --- | --- | --- |
| Frequency, n (%) | 72 (16.5) | 313 (71.6) | 48 (11.0) | 4 (0.9) | 437 (100) |
| Median age (years), IQR | 39.9 (30.9-49.3) | 35.7 (30.5-43.0) | 38.0 (31.6-42.5) | 36.8 (34.3-37.9) | 36.1 (30.8-43.9) |
| Female, n (%) | 40 (55.6) | 159 (50.8) | 29 (60.4) | 2 (50.0) | 230 (52.6) |
| Median CD4 count (cells/mm^3^), IQR | 89.5 (29.0-224) | 56 (18.0-112) | 93.0 (53.8-182) | 88.0 (26.8-137) | 62.0 (22.5-133) |
| On ART, n (%) | 28 (38.9) | 95 (30.4) | 19 (39.6) | 0 (0) | 142 (32.5) |
| Mortality at 12 weeks, n (%) | 11 (15.3) | 62 (19.8) | 9 (18.8) | 1 (25.0) | 83 (19.0) |

Abbreviations: ART = antiretroviral therapy; IQR = interquartile range; LAM = lipoarabinomannan.

*This group were excluded from laboratory analyses

**Supplementary Table S2 Participant characteristics by Mycobacterium tuberculosis blood stream infection and vital status**

P values by Fisher’s Exact Test; **given for *on ART* versus *ART naïve* or *defaulted.*

ART = Antiretroviral therapy (ART); Mtb BSI = *Mycobacterium tuber*culosis blood stream infection; Q1, Q3 = First quartile, third quartile

**Supplementary Table S3 Matrix metalloproteinase and extracellular matrix breakdown product concentrations**

|  | **No TB** | | **Confirmed TB** | | **Clinical TB** | | p values^$^ | |
| --- | --- | --- | --- | --- | --- | --- | --- | --- |
| Plasma | Median | Q1, Q3 | Median | Q1, Q3 | Median | Q1, Q3 | No TB vs Confirmed TB | No TB vs Clinical TB |
| MMP-1 (pg/ml) | 4477 | 1988, 7640 | 5316 | 2686, 9756 | 5163 | 2671, 9799 | 0.112 | 0.162 |
| MMP-3 (pg/ml) | 12840 | 8180, 22951 | 10716 | 6934, 15935 | 8535 | 5528, 14080 | **0.021** | **0.006** |
| MMP-7 (pg/ml) | 1071 | 455, 1967 | 712 | 330, 1406 | 525 | 174, 1589 | 0.046 | 0.088 |
| MMP-8 (pg/ml) | 10602 | 2019, 32205 | 23712 | 7688, 47571 | 4430 | 2156, 27099 | **0.003** | 0.324 |
| MMP-9 (pg/ml) | 26512 | 11079, 45181 | 26574 | 14400, 58120 | 23853 | 12909, 50802 | 0.220 | 0.667 |
| MMP-10 (pg/ml) | 7809 | 4990, 11089 | 5807 | 4147, 8009 | 5310 | 3798, 7897 | **<0.001** | **0.001** |
| PIIINP (pg/ml) | 21380 | 14323, 41866 | 22712 | 13978, 40724 | 19308 | 12082, 28705 | 0.941 | 0.193 |
| HA (ng/ml) | 8.26 | 5.24, 12.9 | 8.287 | 5.815, 10.88 | 8.815 | 5.29, 15.5 | 0.640 | 0.872 |
| Col4⍺1 (ng/ml) | 7.98 | 7.45, 9.65 | 9.81 | 8.42, 12.0 | 11.7 | 7.87, 14.7 | 0.017 | 0.103 |

Confirmed TB*: Mycobacterium tuberculosis* identified in clinical samples; Clinical TB*:* TB was likely and the patient was treated for TB but no microbiological confirmation was obtained; No TB: TB was excluded on clinical and microbiological grounds. Abbreviations: Col4⍺1 = collagen IV alpha 1; HA = Hyaluronic Acid (HA); Q1,Q3 = first quartile, third quartile; MMP = matrix metalloproteinase; PIIINP = procollagen N-terminal propeptide. HA and Col4⍺1 measurement were on a random subgroup of 73 participants.

^$^A Bonferroni correction for multiple comparisons indicated that a p value of <0.025 was equivalent to a significance threshold of <0.05. Significant p values are shown in bold.

**Supplementary Table S4 Matrix metalloproteinase and procollagen III N-terminal propeptide concentration by sex**

P value by Mann Whitney U or Fisher’s Exact test. MMP = matrix metalloproteinase; PIIINP = procollagen N-terminal propeptide; Q1,Q3 = first quartile, third quartile.

Supplementary Figure S1 Plasma matrix metalloproteinase (MMP) and matrix-derived biomarkers by diagnostic category in male and female participants

Plasma matrix metalloproteinases and matrix-derived biomarkers measured in hospitalised participants with HIV infection and symptoms suggestive of TB (either subsequently microbiologically confirmed or otherwise clinically diagnosed) or in hospitalised HIV positive participants with symptoms due to other diagnoses (no TB), demonstrating similar findings in male and female participants. No statistical tests are reported. PIIINP = procollagen III N-terminal propeptide.


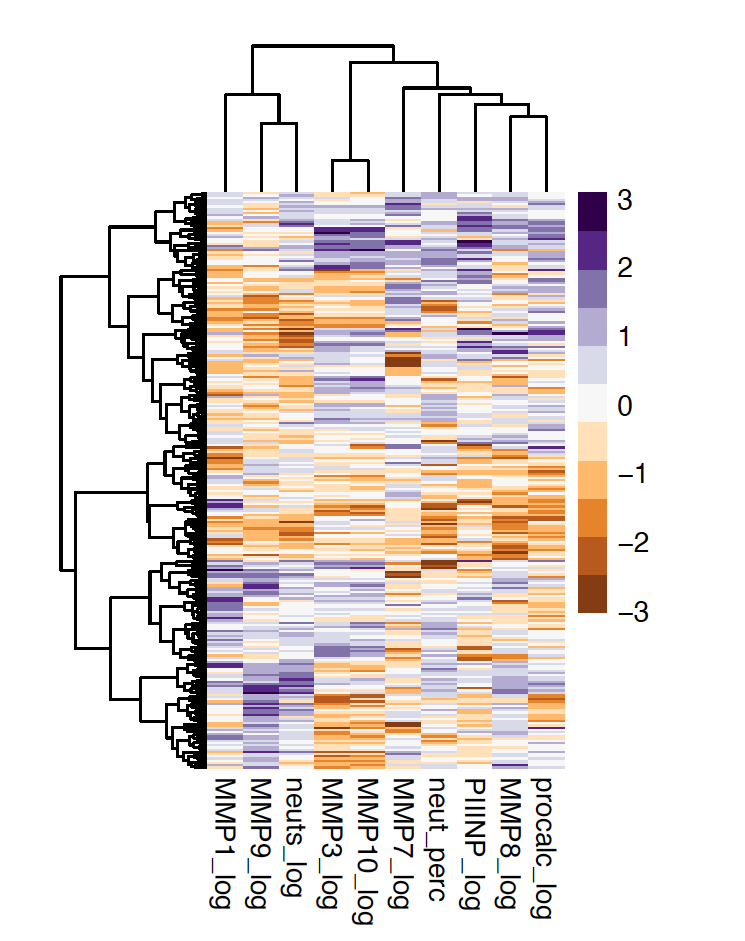


**Supplementary Figure S2 Plasma matrix metalloproteinase-8 associates with collagen turnover and acute inflammation**

Hierarchical clustering analysis demonstrates that plasma matrix metalloproteinase-8 (MMP-8) most closely associated with procalcitonin (procalc) concentrations. Plasma MMP-3 and MMP-10 clustered together, whilst MMP-9 mostly closely clustered with neutrophil count (neuts). The analysis was on scaled data, excluding extreme outliers, which were less than ten data points for any one analyte. Neuts_perc = neutrophil percentage; PIIINP = procollagen III N-terminal propeptide.
